# A Novel Enterococcus Phage Endolysin Lys22 with a Wide Host Range Against Mixed Biofilm of *Enterococcus faecalis*, *Staphylococcus aureus*, and *Acinetobacter baumannii*

**DOI:** 10.3390/pathogens14101060

**Published:** 2025-10-20

**Authors:** Ziqin Yang, Xue Du, Nannan Hu, Meng-Ai Feng, Jiaoyang Xu, Hailin Jiang, Na Zhang, Honglan Huang, Jinghua Li, Hongyan Shi

**Affiliations:** 1 Department of Pathogenobiology, College of Basic Medical Science, Jilin University, Changchun 130021, China; yzq1282251516@163.com (Z.Y.); 18737337090@163.com (N.H.); mengai0426@163.com (M.-A.F.); xujiaoyang2025@163.com (J.X.); jianghl525@163.com (H.J.); hhl@jlu.edu.cn (H.H.); ljh@jlu.edu.cn (J.L.); 2 Affiliated Hospital of Changchun University of Chinese Medicine, 1478 Gongnong Avenue, Chaoyang District, Changchun 130021, China; duxue-22@163.com; 3 Department of Genetics, Qujing Maternal and Child Health-Care Hospital, Qujing 655000, China; zn13844644056@163.com

**Keywords:** endolysin, composite biofilms, multidrug resistance, *Staphylococcus aureus*, *Enterococcus faecalis*, *Acinetobacter baumannii*

## Abstract

The global surge in multidrug-resistant (MDR) bacterial pathogens has created an urgent imperative for innovative antimicrobial strategies. *Enterococcus faecalis*, *Staphylococcus aureus*, and *Acinetobacter baumannii* demonstrate remarkable antibiotic resistance and dominate hospital-acquired infections. These bacteria often form biofilms, a complex community structure that shields them from immune system phagocytosis, resists antibiotic penetration, and enhances their survival in hostile environments. In clinical cases, these bacteria often form mixed biofilms and lead to treatment failures. Phages and their derivatives have emerged as promising candidates in the fight against drug-resistant bacteria. Lys22, an endolysin derived from an enterococcus phage, has been cloned and demonstrated to possess a broad host range, effectively targeting *E. faecalis*, various *Staphylococcus* species, and *A. baumannii*. When applied to the biofilms formed by these bacteria, Lys22 was found to significantly inhibit both simple and complex biofilms in vitro. Virulent genes, including *agrA*, *sarA*, and *icaA* in *S. aureus*; *asa1*, *cylA*, and *gelE* in *E. faecalis*; and *OmpA* and *lpsB* in *A. baumannii* were also downregulated by Lys22. Notably, Lys22 also exhibited a robust protective effect against dual or triple infections involving *E. faecalis*, *S. aureus*, and *A. baumannii* in a zebrafish embryos model, highlighting its potential as a therapeutic agent in combatting multi-bacterial infections.

## 1. Introduction

Microorganisms may exist in a free-living state or as alliances with different or identical species [1]. An ordered arrangement of multicellular colonies forming on biological or non-biological surfaces and liquid interfaces is termed a biofilm [2].

*Enterococci*, *Staphylococcus aureus*, and *Acinetobacter baumannii* are priority members of resistant bacteria that need to explore effective new antimicrobial agents [3]. *Enterococci* are very important pathogens in nosocomial infections, and *E. faecalis* occupies about 80% of enterococcal clinical isolates [4]. *E. faecalis* showed strong tolerance to some extreme environments including low pH, high concentration of NaCl, poor nutrition, and some antibiotics. *E. faecalis* has intrinsic and acquired resistance to many antibiotics through encoding low-affinity antibiotic binding proteins, efflux pumps, low membrane permeability, modifying enzymes, ribosome protection proteins, and changes in antibiotic targets [3,5]. Notably, biofilm formation serves as a multifunctional survival strategy of *E. faecalis*, enabling antibiotic evasion, facilitating bacterial dissemination, and acting as a reservoir for resistance gene exchange [6,7].

Similarly, *S. aureus* remains a preeminent global pathogen causing pneumonia, prosthetic joint infections, surgical site complications, and healthcare-associated bacteriemia [8]. The emergence of pan-drug-resistant strains has elevated this pathogen to a critical priority in antimicrobial development [9]. Like *E. faecalis*, its capacity to form tenacious biofilms on both biotic and abiotic surfaces significantly contributes to therapeutic failure in clinical settings [10,11,12].

Among Gram-negative pathogens, *A. baumannii* stands out as a notorious cause of hospital-acquired infections, with its innate multidrug resistance frequently rendering conventional therapies ineffective [13]. Specifically, carbapenem-resistant strains occupy the first place of WHO’s critical priority list for antibiotic development [14]. The pathogen’s extraordinary environmental persistence stems from its remarkable adaptive mechanisms, particularly through the biofilm-mediated colonization of both living tissues and medical devices, thereby establishing protected reservoirs against antimicrobial agents [15,16,17].

Clinically relevant biofilms rarely exist as mono-species communities, with persistent infections frequently involving complex polymicrobial consortia [18]. The frequent coexistence of *E. faecalis* and *A. baumannii* with *S. aureus* in chronic infections exacerbates clinical outcomes, as mixed biofilms not only prolong patient recovery but also accelerate the evolution of drug-resistant variants [19].

Phage-derived endolysins, which lyse bacterial cells by hydrolyzing peptidoglycan bonds during progeny phage release, have shown promise in biofilm eradication [20]. However, their narrow specificity limits their application against polymicrobial infections [21,22].

In this study, we characterized Lys22, a novel endolysin originated from Enterococcus phage LY0322. This enzyme demonstrated broad-spectrum activity against *E. faecalis*, multiple *Staphylococcus* species, *A. baumannii*, *A. pittii*, *A. nosocomialis*, and *Enterobacter hormaechei*. We subsequently evaluated its anti-biofilm efficacy against single-, dual-, and triple-pathogen consortia involved with *E. faecalis*, *S. aureus*, and *A. baumannii* in vitro, and assessed its protective effects in zebrafish embryo infection models challenged with these pathogens.

## 2. Materials and Methods

### 2.1. Bacterial Strains and Zebrafish Embryos

All bacteria used in this study were isolated from the Affiliated Hospital of Changchun University of Chinese Medicine and identified by a Matrix Assisted Laser Desorption Ionization (MALDI)-Time of Light (TOF)-Mass Spectrometer (MS) after purification. Then, 16S rDNA were multiplied and analyzed by routine methods. All 16S rDNA sequences were submitted to NCBI and accession numbers are listed in Table 1. The minimum inhibitory concentration (MIC) of target bacteria was tested according to the Clinical and Laboratory Standards Institute (CLSI)1 method (https://clsi.org/resources (accessed on 3 May 2022)).

Unhatched zebrafish embryos (0–48 hpf) used in this study are from AB strains.

No adult zebrafish were maintained in our laboratory; the experiments were conducted exclusively with fertilized embryos prior to hatching.

### 2.2. The Expression of Lys22

The gene ID of Lys22 in NCBI is 40101561. A gene of Lys22 was cloned into pET28a (+) vector with 6×His label according to the universal method [23]. The plasmid was transformed into E. coli BL21 by the heat shock method. After successful transformation, E. coli BL21 was incubated in 200 mL LB medium containing 0.1 mg/mL of kanamycin and cultured at 37 °C until OD_600_ reached between 0.4 and 0.6. Then, Lys22 expression was induced by 0.1 mM isopropyl β-D-thiogalactoside (IPTG) at 20 °C for 16 h. The supernatant of culture medium was collected by centrifugation at 4 °C, 8000× *g* for 10 min. To remove miscellaneous proteins and a part of endotoxins, the supernatant was filtrated with a 100 KDa ultrafiltration tube (Milipore) at 4 °C and 5000 g for 15 min. The obtained filtrate was further concentrated with a 10 KDa ultrafiltration tube at 4 °C and 5000× *g* centrifugation for 15 min. The concentrated proteins were washed and collected with a Lysis Buffer [24] mmol Tris-HCl (pH 8.0)] and analyzed by SDS-PAGE. Finally, His-tagged Lys22 were purified by Ni-NTA and collected by 40 mM to 250 mM imidazole washing. Concentrations of Lys22 in a series of imidazole elutions were determined by a BCA protein determination kit [25]. Amino acid sequences of expressed Lys22 were performed to analyze their Zn+ binding sites using the SWISS-MODEL server [26].

### 2.3. Determination of the Host Range of Endolysin Lys22

The frozen tubes with bacteria were placed in 37 °C water immediately after being taken out of the −80 °C refrigerator. After rapid thaw, 100 μL of stored bacteria were inoculated into 1 mL liquid BHI and cultured in a shaking incubator at 37 °C, 150 rpm for 1 h. Then, all bacteria were inoculated regionally in a BHI solid medium and cultured overnight to obtain a purified colony at 37 °C. Subsequently, single colonies were selected and inoculated into LB broth and cultured at 37 °C with shaking at 150 rpm until reaching the logarithmic phase (OD_600_ = 0.5–0.6). Then, 100 μL of bacterial culture was evenly inoculated using a sterile spreader to evenly cover LB agar plates. After the surface became dry, 5 μL of endolysin Lys22 was dropped to the surface of the agar, and the plates were incubated overnight at 37 °C. The presence of lysis zones were observed to determine the susceptibility of the strains to Lys22. Lysis zones indicate effective lysis by Lys22, while their absence suggests that the strains are resistant to the lysin [27].

### 2.4. The Stability of Lys22

To evaluate the viability as a potential therapeutic agent, the stability of Lys22 under different conditions was tested according to universal methods with small modification [28,29]. *E. faecalis*, with 16S rRNA accession number MH236318, was used in these experiments.

To test the lytic activity of Lys22 under different concentrations of NaCl or EDTA, freshly cultured *E. faecalis* was suspended in phosphate-buffer saline (PBS, pH 7.4) to a final OD_600_ of 0.5, containing various concentrations of EDTA ranging from 0 to 800 µM or NaCl ranging from 0 to 1000 µM. Then, Lys22 was added into the mixture and incubated 2 h at 37 °C. Finally, the OD_600_ of the mixtures were tested to evaluate the lytic activity of Lys22. Similarly, the lytic activities of Lys22 at different pH values (pH = 3~13) were also measured using a universal method [30].

To evaluate the thermal stability of Lys22, Lys22 was treated at different temperatures including 25 °C, 37 °C, 45 °C, 55 °C, 65 °C, and 75 °C for 30 min. After treatment, Lys22 was added into PBS-suspended *E. faecalis*. The killing effect of Lys22 against *E. faecalis* was tested by measuring the decrease in OD_600_ within 100 min [29].

To evaluate the effect of serum on the bactericidal activity of endolysins, *E. faecalis* cells suspended in PBS buffer containing various concentrations of serum (2, 10, 20, and 50%) were treated with 50 μg/mL endolysins for 1 h at 37 °C [30].

In all above tests, the final concentration of Lys22 was adjusted to 50 µg/mL according to MIC tests (Supplementary Figures S3 and S4). All experiments were repeated three times.

### 2.5. The Effect of Lys22 on Enterococcus Faecalis Biofilm

#### Confocal Laser Scanning Microscopy (CLSM) Analysis

In a 24-well plate (BeyoGold™ 24 Well Cell Culture Plates, Beyotime Biotechnology, Shanghai, China), we added a suspension of *E. faecalis* cultured to the logarithmic phase, followed by Lys22 to achieve an intermediate concentration of 50 μg/mL. After incubating the 24-well plate at 37 °C for 6 h, the biofilm in each well was washed with 1 mL 0.01 M PBS and stained by a Bacterial Viability Kit (Molecular Probes)( Thermo Fisher Scientific, Waltham, MA, USA) according to attached procedures. Following another washing step with 0.01 M PBS, the stained biofilm was air-dried for 10 min and fixed with 1 mL of 2.5% glutaraldehyde (Solarbio, Beijing, China) for 30 min at room temperature. Finally, we washed the biofilm again with 0.01 M PBS and analyzed it using a Leica Ultra View VOX confocal laser-scanning microscope (Leica Microsystems, Wetzlar, Germany), as described in a previous paper [31].

### 2.6. Inhibition of Biofilm in Human Dentin Slices by Lys22

Briefly, the fully developed teeth were obtained from the Stomatology Hospital of Jilin University. The crown was removed using a gold needle, after which the dentin was cut into sections measuring 5 mm × 5 mm × 1 mm. Once the dentin sections were obtained, they were autoclaved at 121 °C for 15 min, as described in a previous paper [32].

To study the impact of Lys22 on the inhibition of biofilm formation, *E. faecalis* was inoculated onto sterilized detin slices in the presence or absence of Lys22 at a concentration of 50 μg/mL, and then cultured for 6 h at 37 °C in a 24-well plate. After incubation, the dentin slices were washed with 0.01 M PBS three times, air-dried at room temperature, and fixed with 2.5% glutaraldehyde for 30 min. Then, the buffer was replaced with increasing concentrations of ethanol (70%, 80%, 90%, 95%, and 100% for 15 min each) to dehydrate the slices. Finally, the dried slices were sprayed with gold and observed under a field-emission scanning electron microscope (FESEM, JSM-6700F, JEOL, Tokyo, Japan, 8.0 KeV), as described in previous papers [28].

To study the effect of Lys22 on mature biofilms, *E. faecalis* was inoculated onto sterilized dentin slices and cultured for 6 h at 37 °C in a 24-well plate first. Next, the plate was washed with 0.01 M PBS and the medium in the experimental wells was replaced with 1 mL of BHI medium containing Lys22 at a concentration of 50 μg/mL. The dentin slice was then treated and observed using the same method as described above.

### 2.7. The Effect of Lys22 on Mixed Biofilms

#### 2.7.1. Turbidity Test and Crystal Violet Method

An excerpt from *E. faecalis* used in the upper experiments, which has a 16S rRNA accession number of MH236318. Methicillin- and vancomycin-resistant *S. aureus* and imipenem-resistant *A. baumannii* were selected as host bacteria in this experiment and subsequent experiments. The 16S rRNA accession numbers of *S. aureus* and *A. baumannii* are OK642793 and PP660341, respectively. All bacteria were overnight cultured in a BHI solid medium at 37 °C. The single colonies were inoculated into BHI broth and cultured at 37 °C with shaking at 150 rpm for 6 h until reaching the logarithmic phase (OD_600_ = 0.5–0.6). Then, concentrated Lys22 (500 μg/mL) was mixed into bacterial cultures and adjusted its working concentration to 50 μg/mL. Subsequently, the mixture was added into 96-well plates at 200 μL per well and incubated at 37 °C. The planktonic bacteria and biofilm were tested at 6, 12, and 24 h. For planktonic bacterial evaluation, the culture media were gently absorbed out and added into new 96-well plates to test OD_600_ (BioTek Instruments, Winooski, VT, USA). For biofilm evaluation, the plates were washed three times with 200 μL of 0.1 M PBS and air-dried at room temperature after culture media were removed. Subsequently, 200 μL of 0.1% crystal violet was added into each well and kept for 10 min. Then, the solutions were taken out, and the wells were gently washed three times with 0.1 M PBS and air-dried at room temperature. Finally, 200 μL of 95% ethanol was added to each well and maintained over 10 min to release the crystal violet combined in the biofilms. The OD_570_ of released crystal violet was tested by a microplate spectrophotometer to quantify biofilm content [33]. The experiments were repeated three times with at least three parallel wells in each group.

#### 2.7.2. Scanning Electronic Microscope Observation

*S. aureus*, *E. faecalis*, and *A. baumannii* were cultured to logarithmic phase (OD_600_ = 0.5–0.6) in 5 mL of LB or BHI medium at 37 °C and 150 rpm. The concentration of Lys22 was adjusted to 50 μg/mL by liquid LB and BHI medium. The cultures were centrifuged, resuspended in equal volumes of the respective medium, and then 500 μL of each bacterial culture was mixed with an equal volume of 50 μg/mL Lys22. This mixture was added to each well of a 24-well plate, ensuring an initial inoculum of 10^8^ CFU for each bacterium; the wells were covered with a sterile glass sheet of 14 mm diameter. For controls, only the bacterial culture was added. The plates were incubated for 6 h, non-adherent cells were removed by aspiration, and the wells were washed thrice with sterile PBS. The biofilms were then freeze-dried and examined using scanning electron microscopy (SEM) (JEOL Ltd., JSM-7900F, 1-2 Musashino 3-chome, Akishima, Tokyo, Japan) [34].

In addition to scanning electron microscopy observations, we also performed viable bacterial counts on the surviving bacteria from the glass slides. The slides were resuspended in 0.1 M PBS solution, and bacterial cells were completely detached into the buffer through pipetting and washing. After 10-fold serial dilution with 0.1 M PBS, the suspensions were inoculated onto BHI solid medium and incubated overnight at 37 °C in an inverted incubator. Finally, individual colonies on the medium were classified and counted based on the colony characteristics of different bacteria.

### 2.8. Quantitative Real-Time PCR (qRT-PCR)

*S. aureus*, *E. faecalis*, and *A. baumannii* were inoculated into 50 mL of LB broth and incubated at 37 °C with shaking at 150 rpm for 6 h and adjusted the bacteria concentration to 10^8^ CFU/mL. Subsequently, Lys22 was added into cultures at working concentrations of 50 μg/mL. The same volume of ddH_2_O was added to the control cultures. Then, the cultures were cultured for 6 h again in the same conditions. To prevent RNA degradation, RNase inhibitor (Thermo Fisher Scientific, Waltham, MA, USA) was added, and cells were immediately cooled in a dry ice–ethanol bath (95% ethanol) for 30 s. Cells were then harvested by centrifugation at 12,000 rpm for 1 min at 4 °C, and total RNA was isolated using the Total RNA Kit (Yisheng Biotechnology, Shanghai, China). A real-time quantitative reverse transcription–polymerase chain reaction (qRT-PCR) was employed to analyze the transcript levels of virulence genes, including agrA, aur, hla, hld, icaA, sarA, and sigB in *S. aureus*; efa, ace, esp, ebp, cylA, gelE, and asa1 in *E. faecalis*; and ompA, bfmS, abaR, csuC, lpsB, PbpG, and plcD in *A. baumannii* (Supplementary Table S1). qRT-PCR reactions were performed using SYBR Green Premix (Yaenzyme Biotechnology, Nanjing, China) and a real-time fluorescent quantitative PCR system ( Thermo Fisher Scientific, Waltham, MA, USA). Gene-specific primers were used, and 16s rRNA served as a housekeeping gene (Supplementary Table S1) to standardize the quantification of target gene expression [35].

### 2.9. Protection of Lys22 to Zebrafish Embryos

The single colony of *E. faecalis*, *S. aureus*, and *A. baumannii* were streaked out and cultured in LB or BHI broth at 37 °C, 120 rpm to the logarithmic period (OD_600_ ≈ 0.6). Then, bacteria were centrifuged for 10 min at 9000 rpm (Hettich Universal 320/320R centrifuge) and resuspended in a zebrafish embryos culture medium (Fishbio, Wuhan, China). Then, the bacterial solution was series diluted to 10^6^, 10^7^, 10^8^, and 10^9^ CFU/mL with PBS. A zebrafish-specific culture medium and unhatched zebrafish embryos were added into 6-well plates with 6 mL medium and 30 embryos per well. Then, different volumes of the prepared bacterial solution were added into the 6-well plates. Subsequently, all plates were cultured at 28 °C for 24 h to test the minimal lethal dose (MLD) of each strain. Once the MLD had been determined, this concentration of bacteria was used as the infective inoculum (challenge dose). To investigate the protection effect of Lys22, Lys22 was added into each well of the zebrafish embryo culture plates shortly before performing the MLD of *E. faecalis*, *S. aureus*, and *A. baumannii*. The living zebrafish embryos were observed and calculated at 4 h, 8 h, 12 h, 15 h, 18 h, 22 h, and 24 h after bacterial challenge. To ensure the death of zebrafish embryos were induced by bacteria attack, a blank control without pathogens nor endolysin Lys22 and a negative control with endolysin Lys22 only were also performed at same time [36,37,38]. The survival of zebrafish larvae was assessed based on the cessation of heart beats and blood circulation. Larvae showing no cardiac activity or blood flow, and lacking any response to gentle mechanical stimulation, were considered dead. The above experiment was repeated three times.

### 2.10. Statistical Analysis

The number of replicates for the assays is provided above, with results presented as means ± standard deviations. Statistical analysis was carried out using one-way ANOVA followed by Dunnett’s test, utilizing SPSS version 23 (SPSS Inc., Chicago, IL, USA). A *p* value of <0.05 was considered significant, and asterisks denote significant differences between treated and untreated groups. For survival experiments, the statistical method used was the Log-rank (Mantel–Cox) test.

## 3. Results

### 3.1. Clone and Expression of Lys22

**Figure 1:**
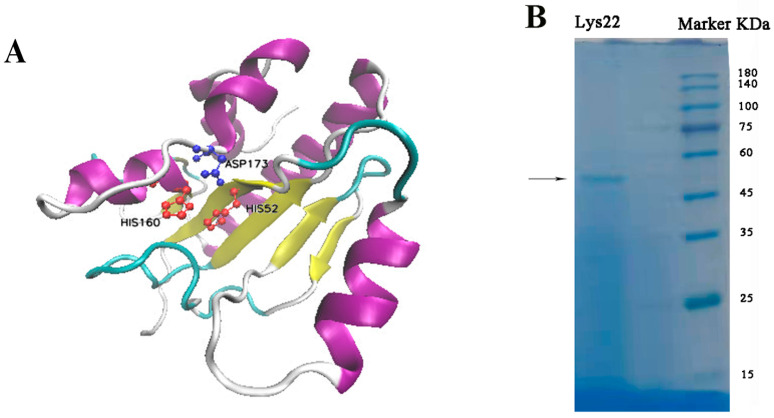
Structure of Lys22 and a 3D construction of Lys22 (**A**), which was performed in the online SWISS-MODEL server. (**B**) The SDS-PAGE result, and the arrow labeled protein is Lys22.

The endolysin from phage LY0322 is an N-acetylmuramoyl-L-alanine amidase comprising 331 amino acids (Protein ID: YP_009624708.1). Structurally, the N-terminal region (residues 46–211) contains a conserved CwIA domain characteristic of peptidoglycan hydrolases that maintain the equilibrium between cell wall synthesis and hydrolysis in Gram-positive bacteria, as documented in *C. difficile* and *Bacillus thuringiensis* [39,40]. Critical zinc-binding residues (His52, His160, and Asp173) form a catalytic triad within the three-dimension structure (Figure 1A). The C-terminal ZoocinA-TRD (target recognition domain) features a β-sheet architecture with antiparallel strands and a short α-helix, mediating species-specific substrate binding. In SDS-PAGE, purified Lys22 is located between marker bands 45 KDa and 60 KDa (Figure 1B).

### 3.2. Host Range of Endolysin Lys22

Primarily, the host range of Lys22 was investigated in Gram-positive bacteria, 36 strains of *E. faecalis* and 49 strains of staphylococci were used as target bacteria in dropping lawn tests. Lys22 demonstrated broad lytic activity to 11 strains of *E. faecalis* and 24 strains of staphylococci (Table 1).

As some endolysins also showed lytic activity to Gram-negative bacteria, 44 strains of *A. baumannii*, 7 strains of *A. pittii*, 1 strain of *A. nosocomialis*, 2 strains of *E. hormaechei,* 20 strains of *Pseudomonas aeruginosa*, and 21 strains of *Klebsiella pneumoniae* were used as target bacteria of Lys22. Among them, 17 strains of *A. baumannii,* all *A. pittii and A. nosocomialis*, and 1 strain of *E. hormaechei* could be lysed by Lys22 (Table 1) [27]. The phylogenetic tree of the 16S rRNA gene sequences of these host bacteria is shown in Supplementary Figure S2.

### 3.3. Stability of Lys22

**Figure 2:**
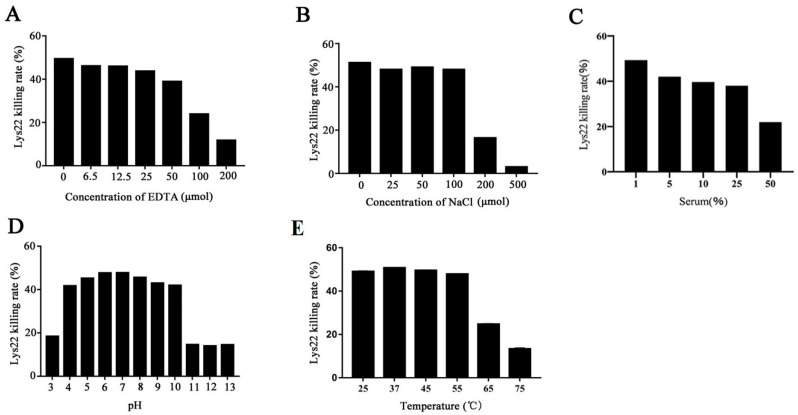
Effects of different factors on Lys22 killing activity on *E. faecalis*. (**A**–**C**) Represent the effect of different concentrations of EDTA, NaCl, and serum on Lys22 killing activity. (**D**,**E**) Represent the effect of different pH and temperatures on Lys22 killing activity.

The stability of Lys22 under different conditions was investigated. When Lys22 was treated with EDTA, its killing effect remained relatively stable at concentrations ranging from 6.5 µM to 100 µM EDTA, as shown in Figure 2A. The study also found that Lys22 retains stable killing effects in a concentration range of 25 µM to 100 µM NaCl, as shown in Figure 2B. The bactericidal activity of Lys22 in the presence of serum is shown in Figure 2C. This suggests that Lys22 is effective in saline environments, which is particularly relevant for its potential application in wound care. The lytic activity of Lys22 also remains stable across a wide range of pH values, from pH 4 to pH 10 (Figure 2D). Finally, the study found that Lys22 exhibits high activity below 55 °C (Figure 2E).

### 3.4. The Effect of Lys22 on E. faecalis Biofilm

**Figure 3:**
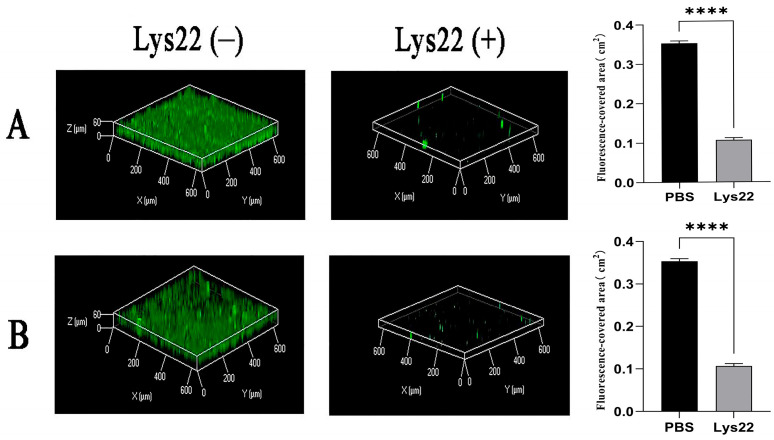
Effects of Lys22 on biofilms of *E. faecalis* under CLSM. Panel (**A**) illustrates the effects of Lys22 administration during the initial stages of biofilm formation, while Panel (**B**) demonstrates the inhibitory impact of Lys22 on mature biofilms. Surviving bacteria within the biofilm were labeled with green fluorescence, and the *Z*-axis represents biofilm thickness. In both Lys22-treated groups, the *Z*-axis heights could not be measured due to the complete disruption of the biofilms, indicating that Lys22 significantly inhibits both the formation and growth of biofilms. Additionally, the fluorescence intensity of surviving bacteria in the Lys22-treated groups was markedly reduced, further confirming the potent inhibitory effect of Lys22 on the formation and maturation of *E. faecalis* biofilms. Data are presented as mean ± SD. Statistical significance is indicated as the following: *p* < 0.0001 (****).

Confocal laser-scanning microscopy (CLSM) was used to analyze the three-dimensional structure of *E. faecalis* biofilms with or without Lys22 (Figure 3). The bacteria that survived within the biofilm were stained by green, fluorescent dyes, while the killed bacteria were not. The biofilm formed in the control group was thicker than that in the experimental group with Lys22, whether it was added at early stage of biofilm formation or in mature biofilm. These findings suggest that Lys22 strongly inhibited *E. faecalis* biofilm formation and destroyed mature biofilms.

**Figure 4:**
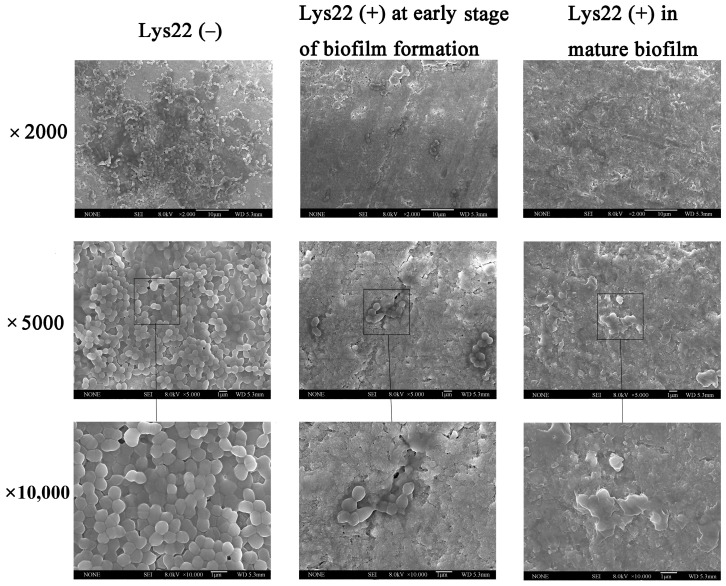
Effects of Lys22 on *E. faecalis* biofilms on dentin tablets. The groups with Lys22 treatment show the inhibition of biofilm not only at an early stage but also in mature biofilms on dentin tablets. For each group, there are three images with 2000×, 5000×, and 10,000× magnifications, respectively.

Then, field-emission scanning electron microscopy (FESEM) was used to analyze the effects of Lys22 on *E. faecalis* biofilms on dentin tablets (Figure 4). The *E. faecalis* biofilm without Lys22 was dense and layered, the bacteria showed normal morphology and clear adhesion to neighbors. However, in the groups treated by Lys22 at an early stage, the number of *E. faecalis* was significantly reduced, the biofilm could not be observed, and the survival bacteria had abnormal morphology. In the group treated by Lys22 in the mature biofilm, except for the debris of lysed bacteria, no biofilm and survival bacterial could be observed.

### 3.5. The Effect of Lys22 Against Mixed Biofilms

**Figure 5:**
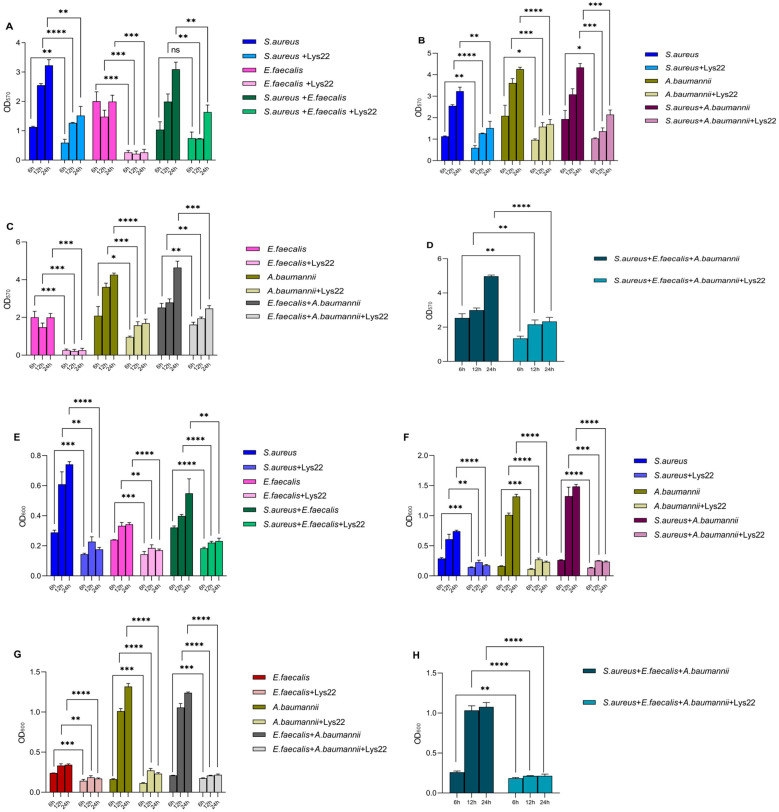
Effect of Lys22 on biofilm formation. The histograms show the effects of Lys22 on biofilm formation (**A**–**D**) and planktonic cell density (**E**–**H**) of *S. aureus*, *E. faecalis*, and *A. baumannii* after 6, 12, and 24 h of treatment. Data are presented as mean ± SD. Statistical significance is indicated as the following: *p* < 0.05 (*), *p* < 0.01 (**), *p* < 0.001 (***), *p* < 0.0001 (****) and ns, not significant.

**Figure 6:**
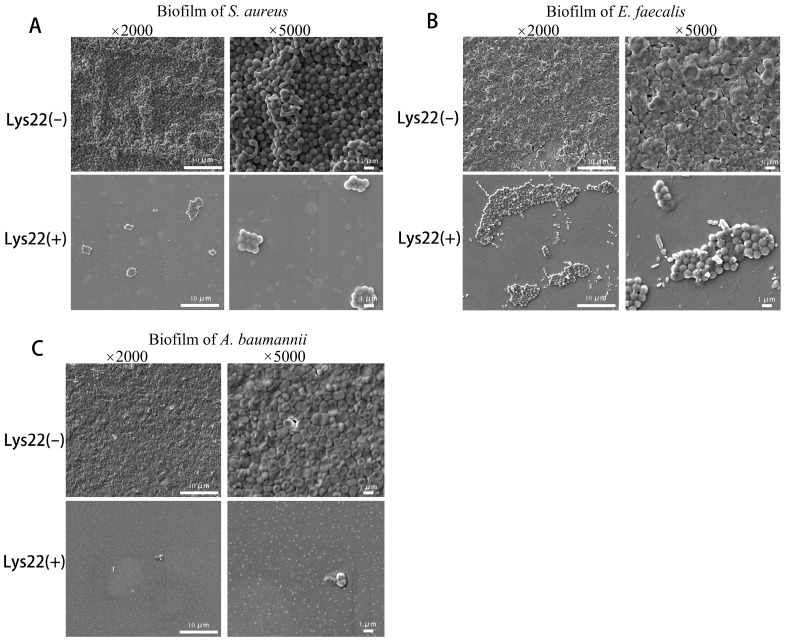
The inhibition of Lys22 to the single biofilms of *S. aureus*, *E. faecalis*, and *A. baumannii* observed under a scanning electronic microscope. In all groups, bacteria were first cultured to the logarithmic phase. Subsequently, Lys22 was added to the Lys22-treated group to achieve a working concentration of 50 μg/mL. The suspensions from each group were then transferred to subsequent groups for continued cultivation for 6 h before they were observed under an electronic microscope. (**A**) is the biofilm of *S. aureus*, (**B**) is the biofilm of *E. faecalis*, and (**C**) is the biofilm of *A. baumannii*.

**Figure 7:**
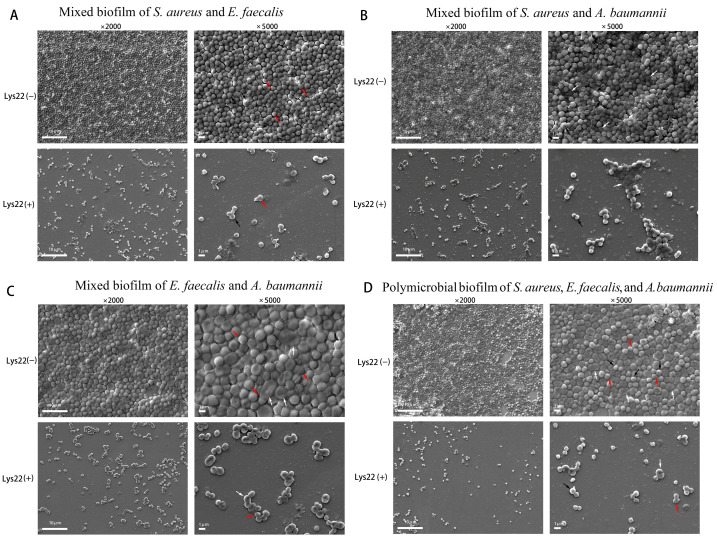
The inhibition of Lys22 to the mixed biofilm of *S. aureus*, *E. faecalis*, and *A. baumannii* observed under a scanning electronic microscope. The method of this test is like that shown in Figure 6. (**A**) is the mixed biofilm of *S. aureus* and *E. faecalis*. (**B**) is the mixed biofilm of *S. aureus* and *A. baumannii*. (**C**) is the mixed biofilm of *E. faecalis* and *A. baumannii*. (**D**) is the mixed biofilm of *S.* aureus, *E. faecalis*, and *A. baumannii*. Black arrows, red arrows, and white arrows, respectively, marked *S. aureus*, *E. faecalis*., and *A. baumannii*. To determine the proportion of viable bacteria in the polymicrobial biofilm, we further performed viable bacterial counts on the polymicrobial biofilm after lysin treatment, with validation based on colony morphology (Supplementary Figure S1).

Gentamicin-resistant *E. faecalis*, methicillin- and vancomycin-resistant *S. aureus*, and imipenem-resistant *A. baumannii* were used as host bacteria to test the inhibition effect of Lys22 on dual- and triple-species biofilms. The biofilm was stained by crystal violet and the optical density of all absorbed crystal violet was tested at 570 nm (Figure 5A–D). Planktonic bacteria were evaluated by testing optical density at 600 nm (Figure 5E–H). The biofilm of all groups was also observed by an electronic microscope (Figure 6 and Figure 7). Compared to untreated controls, Lys22 showed strong inhibition to both dual- and triple-biofilms. The planktonic cells were also effectively inhibited at all tested stages.

### 3.6. Biofilm Removability by SEM

Scanning electron microscopy (SEM) observations unequivocally demonstrated that the administration of Lys22 substantially inhibited biofilm formation in all co-cultured groups (Figure 7), compared to the control groups where only bacterial cultures were used (Figure 6). The bacteria in the groups treated with Lys22 (50 μg/mL) exhibited a marked reduction in forming continuous biofilms. Moreover, most surviving bacterial cells in these groups displayed altered morphology and arrangement compared to control groups. The survival bacteria in the biofilm were also cultured and counted to determine the species according to the morphology of colonies (Supplementary Figure S1).

### 3.7. The Effect of Lys22 on Biofilm-Associated Gene Expression

**Figure 8:**
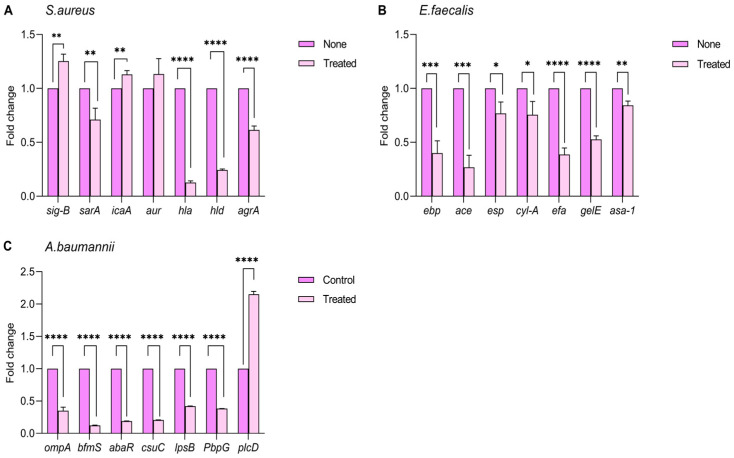
Effects of Lys22 on the virulent gene transcription of *S. aureus* (**A**), *E. faecalis* (**B**), and *A. baumannii* (**C**). All bacteria were cultured in liquid medium with or without Lys22 (50 μg/mL) at 37 °C with 150 rpm shaking for 12 h. Transcriptional profiles were acquired by qRT-PCR. The 16S rRNA was used as a housekeeping gene. Fold changes represent a change in the transcriptions of treated vs. non-treated controls. The experiment was conducted twice, and one gene was tested by three parallel qRT-PCRs each time. Statistical significance is indicated as: *p* < 0.05 (*), *p* < 0.01 (**), *p* < 0.001 (***), and *p* < 0.0001 (****).

The expression levels of selected virulence-associated genes in each bacterial species within the established plankton model were examined. The results demonstrated that Lys22 treatment led to a reduction in the expression of genes related to biofilm formation and pathogenicity in the tested strains. The impact of Lys22 on the genes’ expression was also tested by qRT-PCR. In *S.aureus*, the expression of key pathogenic genes, including alpha-hemolysin (*hla*, *hld*), *sarA*, and *agrA*, were decreased by Lys22 (Figure 8A) (*p* < 0.05). In *E. faecalis*, the plasmid-encoded aggregation factor gene (*asa-1*), cytolysin gene (*cylA*), adherence to collagen gene (*ace*), surface protein gene (*esp*), endocarditis antigen gene (*efa*), gelatinase gene (*gelE*), and enterococcus-binding gene (*ebp*) were significantly downregulated (Figure 8B) (*p* < 0.05). In *A. baumannii*, the outer membrane protein gene (*OmpA*), lipopolysaccharide gene (*lpsB*), penicillin-binding protein gene (*pbpG*), biofilm formation regulator S gene (*bfmS*), Csu pili cluster gene’s member *csuC*, and important component of resistant island *abaR*, were downregulated by Lys22 (Figure 8C). However, *sig-B*, *icaA*, and *aur* of *S. aureus*, and *plcD* of *A. baumannii* were upregulated (*p* < 0.05).

### 3.8. The Protection of Lys22 to S. aureus-, E. faecalis-, and A. baumannii-Attacked Zebrafish Embryos

**Figure 9:**
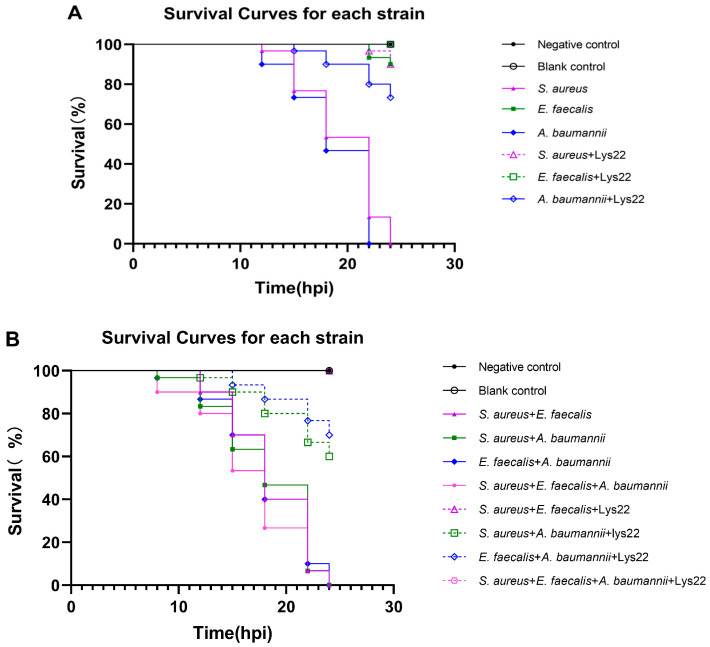
The protective effect of Lys22 on single or mixed-species bacteria-attacked zebrafish embryos. (**A**) shows the survival curves of the single-species-attacked group, and (**B**) shows the survival curves of the groups attacked by mixed species of bacteria. The experiment was conducted three times.

The minimum lethal doses of *S. aureus*, *E. faecalis*, and *A. baumannii* to zebrafish embryos have been experimentally validated as 1 × 10^8^ CFU/well, 1 × 10^8^ CFU/well, and 1 × 10^7^ CFU/well, respectively. The minimum lethal doses were sufficient to induce a 100% mortality rate to zebrafish embryos within 24 h. Lys22 administration dramatically increased the survival rates of zebrafish embryos from the lethal dose of bacterial infections (Figure 9A). The survival rates of the *S. aureus-*, *E. faecalis-*, and *A. baumannii*-infected groups were, respectively, increased to 90%, 100%, and 73.33% by Lys22 treatment (*p* < 0.05). Moreover, Lys22 treatment also increased the survival rates of zebrafish embryos in dual- or triple-species bacterial infections. The survival rates of zebrafish embryos in the *S. aureus* + *E. faecalis* group, *S. aureus* + *A. baumannii* group, *E. faecalis* + *A. baumannii*, and *S. aureus* + *A. baumannii* + *E. faecalis* group were, respectively, increased to 100%, 60%, 70%, and 100% by Lys22 treatment (*p* < 0.05) (Figure 9B).

## 4. Discussion

Endolysins are peptidoglycan hydrolases that degrade the peptidoglycan (PG) layer of the host bacterium ‘from within’ at the end of their lytic multiplication cycle. The journey of endolysin from host bacterial cytoplasm to their PG substrate needs the help of holins. Holins are also a component of the phage lysis cassette of tailed phages. Holins are produced during the late stages of infection and, once a critical concentration is reached, create holes in the cytoplasmic membrane by oligomerization, allowing the endolysins, which have accumulated in the cytoplasm, to access their PG substrate [41]. As Gram-positive bacteria lack outer membranes (OMs), endolysins can easily reach their PG substrate and lyse cell wall when they are exogenously administrated. But the OMs of Gram-negative bacteria form a barrier which prevents exogenous endolysins from accessing and degrading the underneath PG layer, thereby protecting the Gram-negative bacteria from lysin attack. So, overcoming the OM barrier of Gram-negative bacteria became very important to explore the full potential of lysins in the battle against MDR bacteria. Finding lysins with an intrinsic ability to permeate the OM, simultaneously apply lysins with outer membrane permeabilizers (OMPs), and modify the structure of lysins by engineering techniques are currently proposed strategies [42].

Enterococcus phage LY0322 is a lytic phage previously isolated and identified in our lab. The sensitive host bacteria of LY0322 included seven strains of *E. faecalis* and two strains of *E. faecium*. LY0322 cannot lyse any *Staphylococcus* and Gram-negative bacteria. Compared to phage LY0322, Lys22 has an outstanding wider host range, more *E. faecalis* strains, several species of *Staphylococcus* and *Acinetobacter*, and a strain of *E. hormaechei* are sensitive to the lysis of Lys22. But two strains of *E. faecium* sensitive to phage LY0322 were resistant to Lys22. The lytic activity to *Acinetobacter* spp. proved that Lys22 has good intrinsic permeability of the OM of Gram-negative bacteria. Some strains of *Pseudomonas aeruginosa*, *Escherichia coli*, and *Klebsiella pneumonia* were also used as target bacteria in host range tests, but no sensitive strains were found. Interestingly, Lys22 easily produced plaques of sensitive *Staphylococcus* and *Acinetobacter* in a dropping lawn test. Conversely, the plaques in the lawn of different *E. faecalis* are usually small, nontransparent or hard to observe. But in liquid medium, the number of surviving *E. faecalis* both in biofilm and in suspension were dramatically decreased by Lys22. The wide and complicated host range of Lys22 brings us many new issues to explore and understand regarding the mechanisms of lysin–membrane interactions and lysin-peptidoglycan target recognition.

The emergence of multidrug-resistant *S. aureus*, *E. faecalis*, and *A. baumannii* highlights the critical need for alternative therapeutic strategies. These pathogens are notorious for their ability to form robust biofilms on the surface of living or non-living organisms and medical devices, significantly hindering the effectiveness of traditional antibiotics [43]. Lys22 demonstrates significant inhibitory activity against both biofilm formation and mature biofilms of its native host bacterium, *E. faecalis*. Given the significant variations in the time required for mature biofilm formation among different bacterial species, as well as the presence of quorum-sensing competition between bacteria, the colony counting of biofilms at a single time often revealed a disproportionate representation—where one bacterial species dominated while others were significantly underrepresented. Therefore, for mixed-species biofilms, we opted to use Lys22 to interfere with the formation process, since we can evenly introduce each bacterial species at the starting point, thereby ensuring experimental stability and reproducibility. Prior to conducting biofilm-inhibition experiments, we examined the growth pattern of Enterococcus faecalis biofilms through preliminary studies. All subsequent biofilm inhibition experiments were performed based on this established pattern. Details of this experimental section are provided in Supplementary Figure S3.

As *E. faecalis* was detected in 4–40% of primary root canal infections and 24–77% of secondary root canal infections [44]. Root canal therapy is a routine treatment for root canal infections, which can seal and isolate the root canal system from the source of the bacterial infection. However, the failure rate of root canal therapy is as high as 10–15% [45]. The biofilm provides protection for *E. faecalis* from the attack of the environment and facilitates its survival [46,47]. Considering this factor, we tested the effect of Lys22 on the biofilm of dentin slices and found that Lys22 can inhibit the formation of biofilm and has a killing effect on the bacteria in the mature biofilm. Additionally, in dual- or triple-bacteria mixed-culture groups, the biofilms were also inhibited by Lys22.

Moreover, Lys22-induced cell wall destruction also triggered expression changes in some genes related to biofilm formation and pathogenicity. As the qRT-PCR assay showed, in *S. aureus*, *hla*, *hld*, *sarA*, and *agrA* were downregulated, while *sigB* and *icaA* were upregulated (Figure 8A). *Hla* and *hld* are crucial for the pathogenicity of *S. aureus*, and *sarA* facilitates biofilm formation [48,49]. The icaA gene encodes an enzyme, N-acetylglucosamine transferase, which is involved in the synthesis of polysaccharide intercellular adhesin (PIA), a key component of the biofilm in *S. aureus* [50]. The auxiliary gene regulator (*agr*) and alternative sigma factor σB (*sigB*) are regulators. *Agr* is a survival-enhancing gene involved in regulating virulence and intercellular communication genes expression via a two-component signaling system (TCSTS) [51]. In various Gram-positive bacteria, such as *B. subtilis*, *B. cereus*, *L. monocytogenes*, and *S. aureus*, *sigB* is crucial for restoring normal physiological function or balance when bacteria undergo stress, damage, or dysfunction [52]. The upregulation of *sigB* and *icaA* suggests that *S. aureus* activates compensatory mechanisms to survive under lysin suppression.

In *E. faecalis*, *asa1*, *cylA*, *ebp*, *esp*, *efa*, and *gelE*, which are involved in biofilm formation and pathogenesis, were downregulated to varying degrees by Lys22. The product of *asa1* facilitates the adherence to renal tubular cells and human macrophages. The cytolysin gene (*cylA*) encodes a protein capable of lysing both prokaryotic and eukaryotic cells [53]. Additionally, *ebp* and *ace* gene-encoded structures are critical for biofilm formation and adhesion, which play significant roles in experimental urinary tract infections (UTIs) and endocarditis [54]. The surface protein (*esp*), located in the bacterial cell wall, contributes to colonization and biofilm formation. The endocarditis antigen (*efa*) is pivotal in biofilm formation and pathogenesis [55]. The gelatinase gene (*gelE*) is an important factor that hydrolyzes gelatin, casein, and collagen [56]. In *A. baumannii* cells, OmpA promotes host cell apoptosis, epithelial cell adhesion, invasion, biofilm formation, surface motility, and serum resistance [57]. Lipopolysaccharide (*lpsB*) helps evade the host immune response and triggers an inflammatory response [58]. Penicillin-binding protein (*pbpG*) plays a role in peptidoglycan biosynthesis, cell stability, and serum growth [59]. The *abaR* islands are recognized as potential contributors to antibiotic resistance and heavy metal resistance [60].

In *A. baumannii*, virulent genes involved in biofilm formation (*csuC*, *BfmS*, *plcD*), iron acquisition (*IpsB*), encoding outer-membrane proteins (*ompA*), surface glycoconjugates (PbpG), the secretory system (*ompA*, *plcD*), and the quorum-sensing system (*abaR*) were tested in our study [24,61,62,63,64]. All tested genes other than *plcD* were downregulated by Lys22. *PlcD* is very important to *A. baumannii* survival because the product of *PlcD* is implicated in biofilm formation, serum resistance, and antibiotic resistance. Like that of *S. aureus*, a compensatory activation of the protective system may play a role in lysin suppression.

The Lys22 treatment significantly protected zebrafish from lethal infections caused by various bacterial species, including combinations of *E. faecalis*, *S. aureus*, and *A. baumannii*. Lys22 effectively protected zebrafish from MASA infections, with near-complete protection observed. However, its efficacy was reduced against the Gram-negative bacterium *A. baumannii*, potentially due to the presence of bacterial metabolites, resembling lipid A (endotoxin) [65], which may have contributed to host mortality prior to the action of the endolysin. Interestingly, when the three bacteria were co-infected, Lys22 conferred full protection, likely due to inter-bacterial competition in the mixed culture, which reduced the number of pathogenic strains capable of causing zebrafish mortality. Overall, Lys22 demonstrated significant therapeutic potential in in vivo infection models.

## 5. Conclusions

This study confirms that endolysin Lys22 has a wide host range, including *E. faecalis*, *Staphylococcus* spp., and *Acinetobacter* spp., in disrupting biofilms and combating multidrug-resistant bacterial infections. The lytic activity of *Acinetobacter* spp. Proved that Lys22 has good intrinsic permeability to the OM of Gram-negative bacteria.

Lys22’s ability to destroy the single-species biofilm and mixed biofilm of *E. faecalis, S. aureus*, and *A. baumannii* offers a distinct advantage over traditional antibiotics, especially in overcoming polymicrobial biofilm resistance. Lys22 effectively protected zebrafish eggs attacked by the single-, dual- and triple-species attacks of *E. faecalis, S. aureus*, and *A. baumannii.* Additionally, Lys22 effectively downregulated virulent genes such as *agrA* and *icaA* in *S. aureus*; *asa1*, *cylA*, and *gelE* in *E. faecalis*; and *OmpA* and *lpsB* in *A. baumannii*, reducing their pathogenicity.

## Figures and Tables

**Table 1:** Bacteria lysed by Lys22.

Sensitive Bacteria	Accession Numbers of Sensitive Bacteria 16S rRNA
*E* *nterococcus* *faecalis*	MH236308, MH236312, MH236314, MH236328, MH362705, MH236319, MH236320, MH591461 ^△^, MH236341 ^△^, MH236325 ^△^, MH236318 ^△^.
*Staphylococcu* *s* *. aureus*	OK642790 ^◇^, OK642791 ^◇^, OK642793 ^◆^, OK642796 ^◆^, OK642794, OK642795, OK642797, OK642798, OK642799, OK642792.
*Staphylococcus. epidemidis*	OK642800, OK642801, OK642803, OK642804.
*Staphylococcus. haemolyticus*	OK642805, OK642806, OK642807, OK642808, OK642809.
*Staphylococcus. hominis*	OK642810
*Staphylococcus cohnii*	OK642811
*Staphylococcus klosii*	OK642812
*Staphylococcus warneri*	OK642813
*Acinetobacter baumannii*	PP659668, PP659669, PP659670, PP659673, PP660317, PP732465, PP660318, PP660321, PP660327, PP660328, PP732466, PP660333, PP660334, PP660338, PP660341, PP660342, PP660550
*Acinetobacter pittii*	PP660329, PP660332, PP660344, PP660545, PP660551, PP660552, PP659677
*Acinetobacter nosocomialis*	PP660549
*Enterobacter hormaechei*	PP659673

^△^ represents strains that can be lysed by Lys22 but are resistant to phage LY0322. ^◇^-marked strains are methicillin-resistant *S. aureus* (MRSA), while ^◆^-marked strains are resistant to both methicillin and vancomycin.

## Data Availability

All data supporting this study are included in the article and its Supplementary Materials.

## Supplementary Materials

The following supporting information can be downloaded at: https://www.mdpi.com/article/10.3390/pathogens14101060/s1, Figure S1. Results of viable bacteria counts of biofilms; Figure S2. Phylogenetic tree based on the 16S rRNA gene sequences of selected host bacteria; Figure S3. Effect of Lys22 on the biofilm of *E. faecalis* (MH236318); Figure S4. Killing effect of Lys22 at different concentrations of *E. faecalis* (MH236318); Figure S5. Protein standard curve. Figure S6. Lys22 and Phage LY0322 Lysis plaques. Table S1. (a) Primers for the detection of *S. aureus* virulence genes; (b) Primers for the detection of *E. faecalis* virulence genes; (c) Primers for the detection of *A. baumannii* virulence genes.
