# Supplementary material for: A Novel Enterococcus Phage Endolysin Lys22 with a Wide Host Range Against Mixed Biofilm of Enterococcus faecalis, Staphylococcus aureus, and Acinetobacter baumannii
Source: Pathogens. 2025 Oct 20;14(10):1060. doi: 10.3390/pathogens14101060 (PMC12567084; doi:10.3390/pathogens14101060)
Supplement: Supplementary file 1 [file pathogens-14-01060-s001.zip › pathogens-3856446-supplementary.pdf]

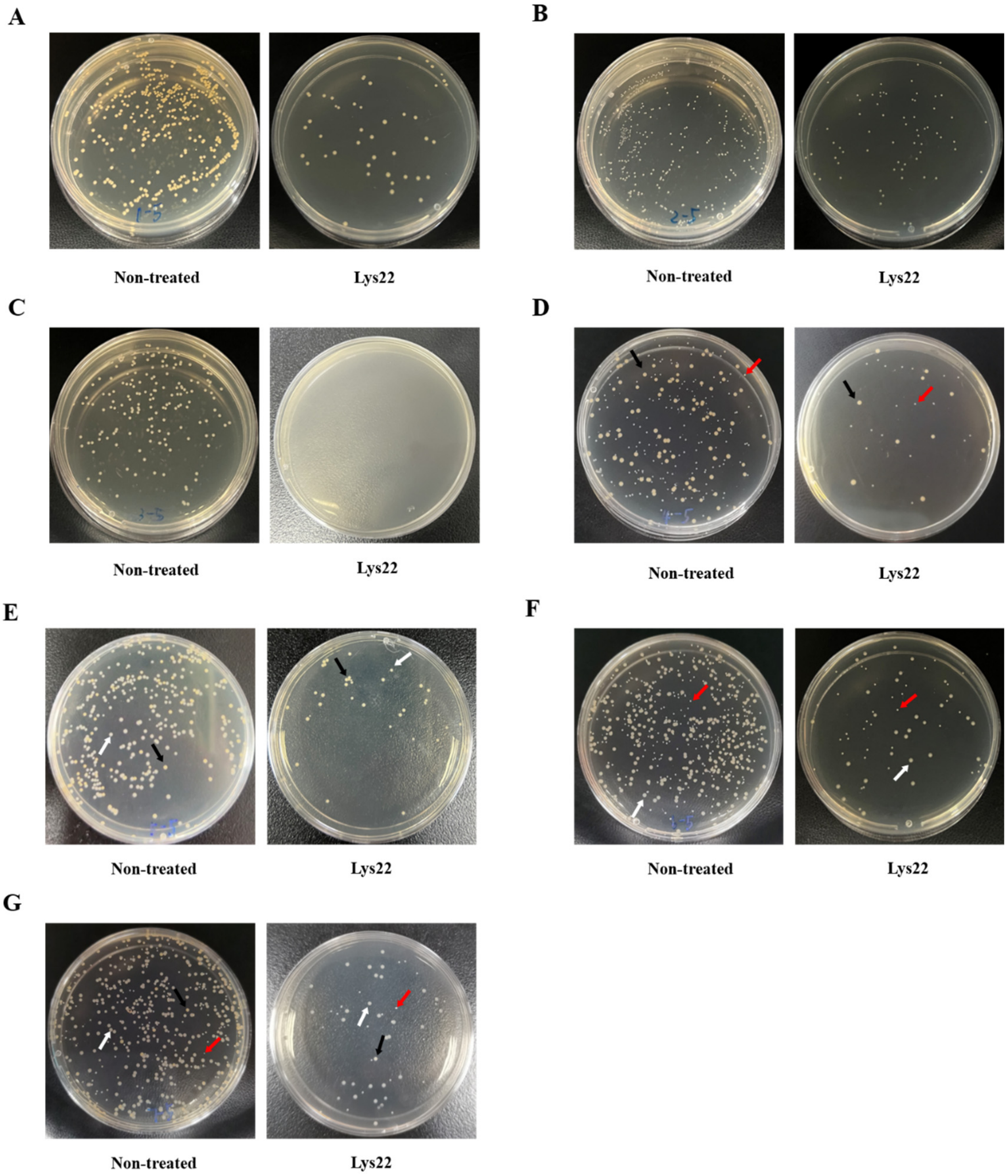

Figure S1. Results of viable bacteria counts of biofilms. Due to the excessive density of bacteria in the original biofilm, a 10-5 concentration of untreated biofilm viable bacteria count plates were obtained by 10-fold multiplicative dilution. In contrast, the Lys22-treated biofilm was used directly for viable bacteria counting experiments using the original concentration. The Photograph of plate count results show bacterial survival in biofilms by *S. aureus* (A), *E. faecalis* (B), *A. baumannii* (C), *S. aureus* and *E. faecalis*（≈1：2） (D),*S. aureus* and *A. baumannii*（≈1：2） (E), *E. faecalis* and *A. baumannii*（≈1:1） (F),and *S. aureus* ,*E. faecalis* and *A. baumannii*(≈1:10:7) (G) after treated for 6h, followed by further treatment with Lys22 (50 μg/mL) or non-treated. Black arrows, *S. aureus*; red arrows, *E. faecalis*; white arrows, *A. baumannii*.

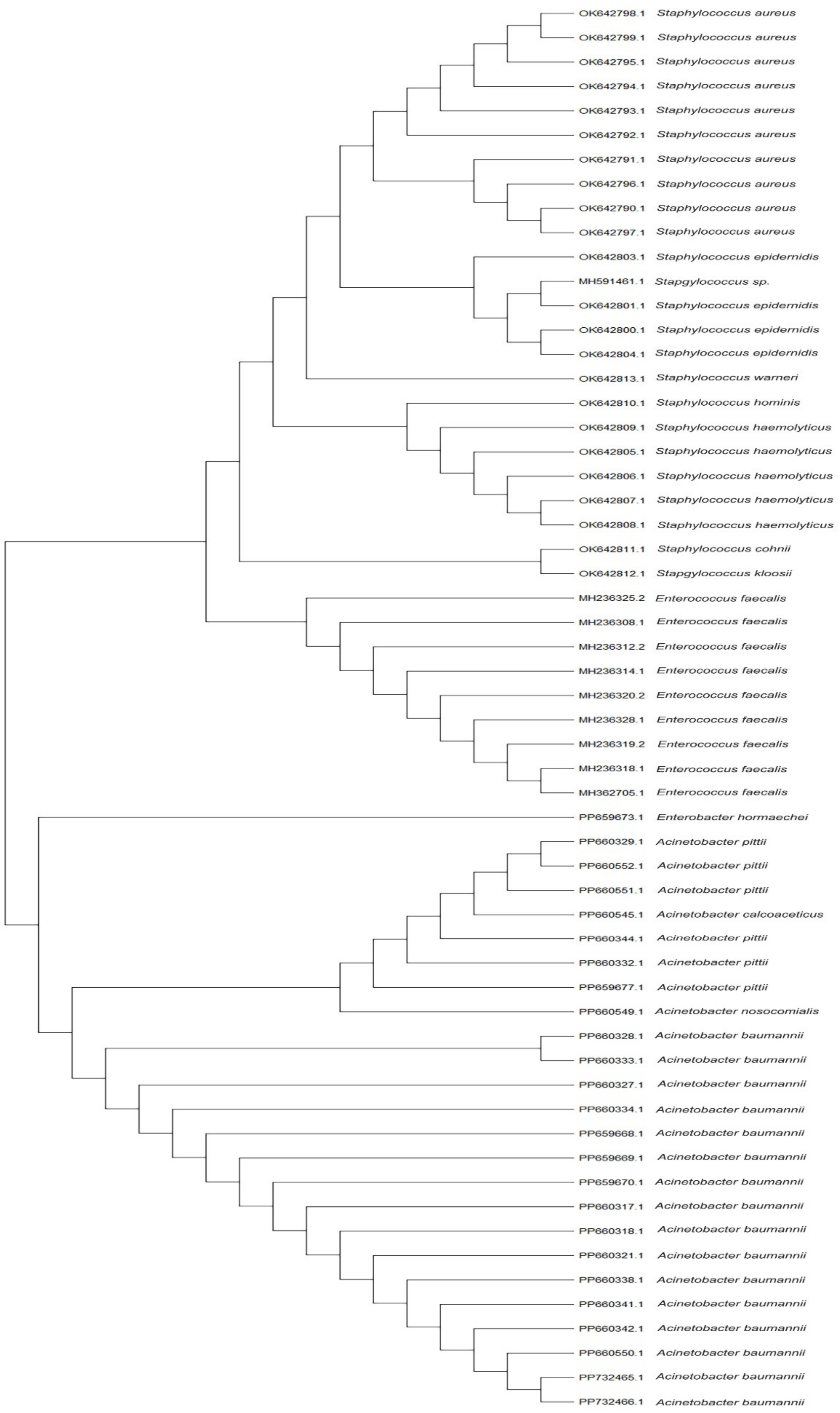

Figure S2. Phylogenetic tree based on the 16S rRNA gene sequences of selected host bacteria. Neighbor-joining cladogram constructed in MEGA11; alignment of the sequences was done with CLUSTALW.

Assay for Lys22 cleavage activity of lyse

The standard curve for protein quantification was established using the BCA protein assay kit. The correlation between protein concentration and absorbance at 562 nm (OD_{562}) was described by the equation y = 0.0009x + 0.1554. Based on this curve, the concentration of the target protein was determined to be approximately 200 μg/mL.

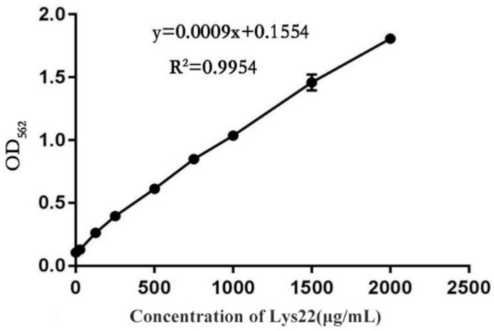

Figure S3. Effect of Lys22 on biofilm of *E. faecalis* (MH236318). The biofilm matrix growth curve of *E. faecalis*.

The turbidity assay results demonstrated that the lytic activity of the enzyme Lys22 exhibited a clear dose-dependent pattern. Under identical initial bacterial concentrations, higher concentrations of Lys22 led to more extensive bacterial lysis, as reflected by the increased transparency of the bacterial suspension. At a concentration of 100 μg/mL, Lys22 almost completely lysed the bacterial cells, whereas even at 25 μg/mL, it showed noticeable bacteriolytic activity, resulting in a significant decrease in the absorbance of the bacterial suspension.

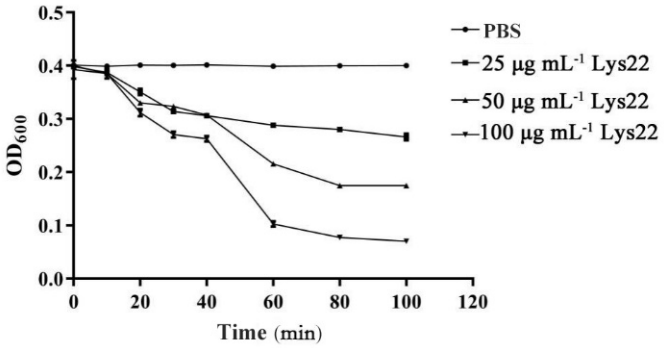

Figure S4. Killing effect of Lys22 at different concentrations on *E. faecalis* (MH236318). With the extension of time and the increase of Lys22 concentration, more *E. faecalis* were killed, OD_{600} of *E. faecalis* culture medium decreased.

Biofilm Matrix Growth Curve

The growth dynamics of the biofilm matrix were evaluated using crystal violet staining, with biofilm biomass quantified by measuring the optical density at 570 nm (OD_{570}). As shown in the figure below, the biofilm biomass of Enterococcus faecalis increased progressively during the first 24 hours of incubation and subsequently declined after reaching its peak. The maximum biofilm formation was observed at 6 hours of incubation.

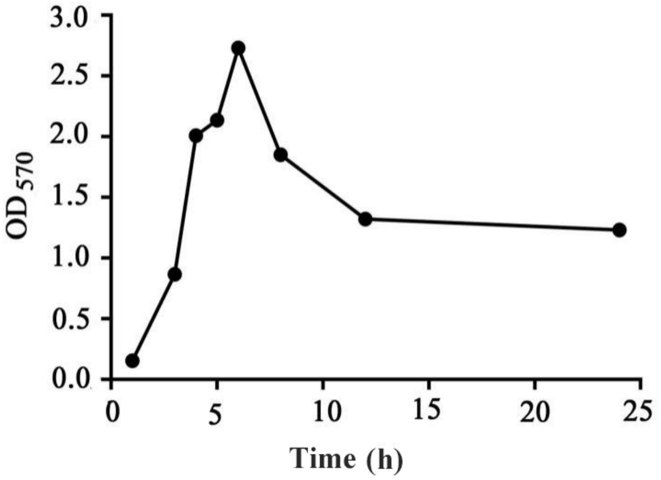

Figure S5. Protein Standard Curve. Growth curve of *E. faecalis* (MH236318) biofilm quantified by OD_{570} after crystal violet staining.

Lys22 and Phage LY0322 Lysis plaques

The lytic activity of the Lys22 lysozyme was evaluated using the plaque assay method. The results are presented in the figure below, which shows the morphology of phage LY0322 on a double-layer agar plate (Figure S6A) and pictures of Lys22 cleavage plaques (Figure S6A).

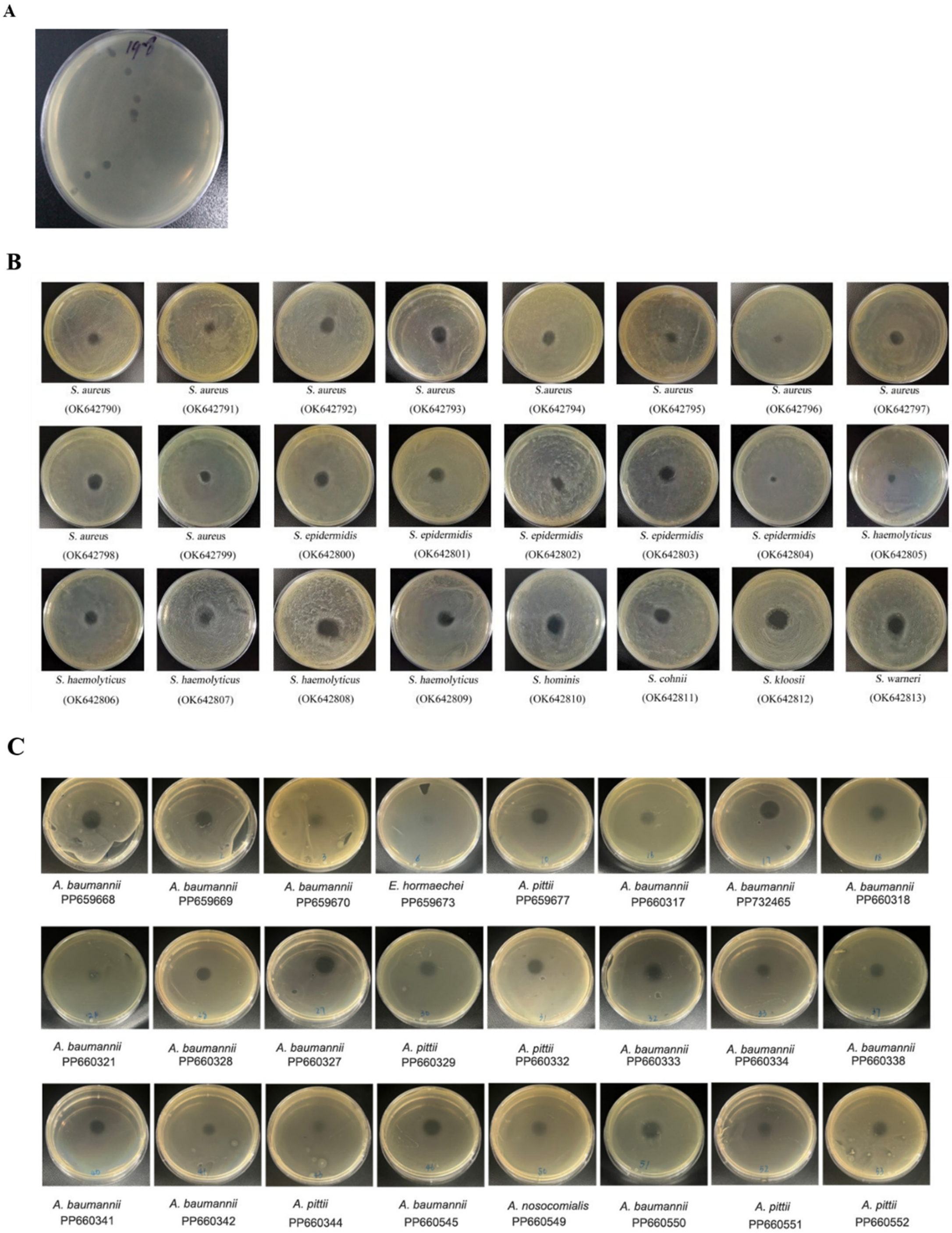

D

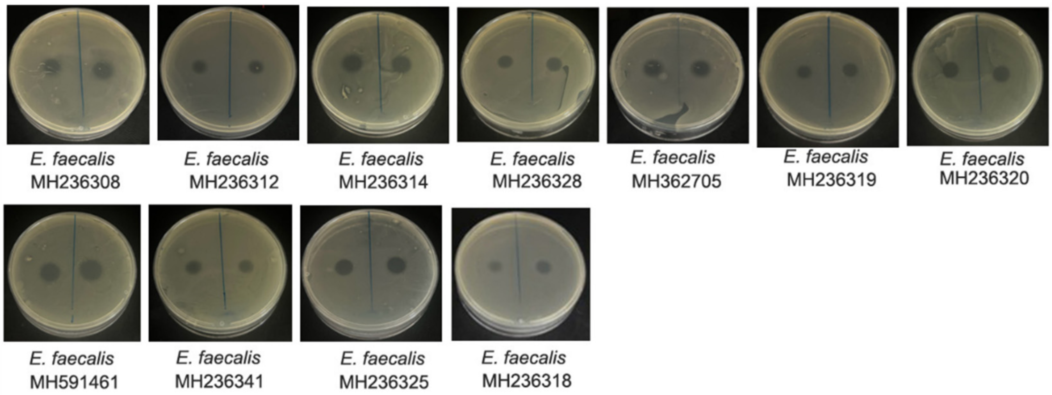

Figure S6. Lys22 and Phage LY0322 Lysis plaques. Morphology of phage LY0322 on a double-layer agar plate(A). The lytic activity of the Lys22 lysozyme was determined using the plaque assay method (B, C and D).

Table S1a. Primers for detection of *S. aureus* virulence genes

Target genes	Primers	Sequence (5′→3′)	Source
16S rRNA	*16S rRNA* F	AGAGTTTGATCCTGGCTCAG	
	16S rRNA R	GGTTACCTTGTTACGACTT	
agrA	*agrA* F	TGA TAA TCC TTA TGA GGT GCT T	WP_431193690.1
	agrA R	CAC TGT GAC TCG TAA CGA AAA	
aur	*aur* F	ACC GTG TGT TAA TTC GTG TGC TA	WP_001225025
	aur R	ATG GTC GCA CAT TCA CAA GTT T	
hla	*hla* F	CGG CAC ATT TGC ACC AAT AAG GC	WP_000857485
	hla R	GGT TTA GCC TGG CCT TCA GC	
hld	*hld* F	ATC GAC ACA GTG AAC AAA TTC AC	WP_001823225
	hld R	CTC TAC TAG CAA ATG TTA CTC AC	
icaA	*icaA* F	TGA ACC GCT TGC CAT GTG	WP_001159427
	icaA R	CAC GCG TTG CTT CCA AAG A	
sarA	*sarA* F	GAG TTG TTA TCA ATG GTC	CAD7354108.1
	sarA R	GTT TGC TTC AGT GAT TCG	
sigB	*sigB* F	AAG TGA TTC GTA AGG ACG TCT	YP_501451.1
	sigB R	TCG ATA ACT ATA ACC AAA GCC T	

Table S1b. Primers for detection of *E. faecalis* virulence genes

Target genes	Primers	Sequence (5′→3′)	Source
16S rRNA	*16S rRNA* F	AGAGTTTGATCCTGGCTCAG	
	16S rRNA R	GGTTACCTTGTTACGACTT	
efa	*efa* F	TGG GAC AGA CCC TCA CGA ATA	WP_002356954
	efa R	CGC CTG TTT CTA AGT TCA AGC C	
ace	*ace* F	GGA GAG TCA AAT CAA GTA CGT TGG TT	WP_010714416
	ace R	TGT TGA CCA CTT CCT TGT CGA T	
esp	*esp* F	AGA TTT CAT CTT TGA TTC TTG G	AAD09858
	esp R	AAT TGA TTC TTT AGC ATC TGG	
ebp	*ebp* F	AAA AAT GAT TCG GCT CCA GAA	WP_154081516
	ebp R	TGC CAG ATT CGC TCT CAA AG	
cylA	*cylA* F	ACT CGG GGA TTG ATA GGC	AAM75253
	cylA R	GCT GCT AAA GCT GCG CTT	
gelE	*gel* F	TAT GAC AAT GCT TTT TGG GAT	WP_002369251
	gel R	AGA TGC ACC CGA AAT AAT ATA	
asa1	*asa1* F	GCA CGC TAT TAC GAA CTA TGA	WP_010785625
	asa1 R	TAA GAA AGA ACA TCA CCA CGA	

Table S1c. Primers for detection of *A. baumannii* virulence genes

Target genes	Primers	Sequence (5′→3′)	Source
16S rRNA	*16S rRNA F*	AGAGTTTGATCCTGGCTCAG	
	16S rRNA R	GGTTACCTTGTTACGACTT	
ompA	*ompA* F	GCTGCTAATGCTGGCGTAAC	WP_000777882
	ompA R	CTACTACAGGAGCAGCAGGC	
bfmS	*bfmS* F	TTGAACTTATTCCACCGCCTTT	WP_000472465
	bfmS R	GCCCGTAATCCGAACTTTGTT	
abaR	*abaR* F	GGTCGAGTCAATCTGCAAAGA	WP_000446779
	abaR R	CTGAGCCCAACCGACATTTA	
csuC	*csuC* F	AAAGCAGGCGAGAAGCATATG	WP_001988023
	csuC R	GGATCGGCAACTCATCTACAATC	
lpsB	*lpsB* F	CAAGCTTGTGGCAAATCCGT	WP_000013434
	lpsB R	CCACCCACAACAACAGCATG	
PbpG	*PbpG* F	AGATGATGAACCGACTCCGC	WP_003384760
	PbpG R	AAAATTCCCAAGTCACGCGC	
plcD	*plcD* F	CAACAACACCGTTTGCGCTA	WP_000079188
	plcD R	TCGCTTTAGTTGCCAGCTCA	
